# *ING4* expressing oncolytic vaccinia virus promotes anti-tumor efficiency and synergizes with gemcitabine in pancreatic cancer

**DOI:** 10.18632/oncotarget.21095

**Published:** 2017-09-20

**Authors:** Yinfang Wu, Xiaozhou Mou, Shibing Wang, Xing-E Liu, Xiaodong Sun

**Affiliations:** ^1^ Department of Hepatobiliary and Pancreatic Surgery, Zhejiang Provincial People’s Hospital, People’s Hospital of Hangzhou Medical College, Hangzhou 310014, P. R. China; ^2^ The Second Clinical Medical College, Zhejiang Chinese Medical University, Hangzhou 310053, P. R. China; ^3^ Clinical Research Institute, Zhejiang Provincial People’s Hospital, People’s Hospital of Hangzhou Medical College, Hangzhou 310014, P. R. China; ^4^ Key Laboratory of Tumor Molecular Diagnosis and Individualized Medicine of Zhejiang Province, Hangzhou 310014, P. R. China; ^5^ Department of Medical Oncology, Zhejiang Hospital, Hangzhou 310007, P. R. China

**Keywords:** oncolytic vaccinia virus, ING4, gene therapy, gemcitabine, pancreatic cancer

## Abstract

With no effective treatments available for most pancreatic cancer patients, pancreatic cancer continues to be one of the most difficult malignancies to treat. Oncolytic virus mediated-gene therapy has exhibited ubiquitous antitumor potential. In this study, we constructed a novel oncolytic vaccinia virus harboring the inhibitor of growth family member 4 gene (VV-*ING4*) to investigate its therapeutic efficacy alone or in combination with gemcitabine against pancreatic cancer cells *in vitro* and *in vivo*. *ING4* expression was determined via quantitative real-time polymerase chain reaction (qPCR) and western blot. The cytotoxicity of VV-*ING4* was measured using a cell proliferation assay. Both flow cytometry and western blot were applied to analyze the cell cycle and apoptosis. Furthermore, the combination inhibitory effect of VV-*ING4* and gemcitabine was assessed using Chou-Talalay analysis *in vitro* and a BLAB/c mice model *in vivo*. We found that VV-ING4 significantly increases *ING4* expression, displayed greater cytotoxic efficiency, and induced pancreatic cancer cell apoptosis and G2/M phase arrest. Additionally, the combination of VV-*ING4* and gemcitabine synergistically effect *in vitro* and *in vivo*. Taken together, our data implicate VV-*ING4* as a conceivable pancreatic cancer therapeutic candidate alone or in combination with gemcitabine.

## INTRODUCTION

Pancreatic cancer is an extremely lethal disease. It is the fourth most prevalent cause of cancer death, with a 5-year survival rate of only 5% [1, 2]. Complete surgical abscission remains the only curative treatment. Sadly, only 10% to 20% of pancreatic tumors are in a position in which they can be surgically removed at the time of diagnosis [3]. For patients with unresectable or metastatic pancreatic tumors, chemotherapy with gemcitabine (Gem) is the first-line therapy. However, acquired resistance to gemcitabine has become an increasing challenge in treating pancreatic tumors [4]. Thus, more potent treatments are needed to improve the outcome of patients with pancreatic cancer.

Gene therapy represents a novel and promising remedial modality for cancer treatment. Gene therapy can be divided into monogene therapy [5] and multigene-based combination therapy [6]. The ING tumor-suppressor family (ING1-5) identified during the past decade act as regulators of transcription, DNA repair, cell cycle checkpoints, apoptosis, angiogenesis, and cellular senescence [7]. ING4, a recently discovered protein, inhibits angiogenesis by correlating with NF-κB subunit p65 (RelA) [8], the loss of contact suppression evoked by MYCN or MYC [9], and the activation of hypoxia inducible factor (HIF) [10]. In addition, substantial studies have reported that ING4 displays pervasive antitumor properties also by inducing apoptosis in a p53-dependent way and significant G2/M cell cycle arrest in a spectrum of cancer cells [6, 8, 11–13]. ING4 also increases cancer cell sensitivity to chemotherapeutic drugs [14, 15]. Conversely, deletion or down-regulation of ING4 is firmly associated with high tumor grade, metastasis, and poor prognosis in a number of cancers, including breast carcinoma [16], glioblastoma [8] and gastric carcinoma [17]. According to the Human Protein Atlas Database (HPA, http://www.proteinatlas.org), ING4 expression is low in pancreatic cancer, indicating its potential functions in transformation and development of pancreatic cancer.

Vaccinia virus (VACV), a member of the poxvirus superfamily, holds several inherent advantages as a vector for cancer gene therapy. Specifically, the virus contains the *TK* gene which codes for a phosphotransferase that enables viral replication. When the gene is disrupted, it inhibits viral replication in normal, non-dividing cells [18]. However, cancer cells have a high concentration of functional nucleotides that enables VACV to selectively replicate in tumor cells. The size of the VACV genome, 200 kb, is permissive to the introduction of foreign genes up to 25 kb in length [19]. Other advantages include an excellent safety profile [20], high capability of transgene expression [21], and the capability to function under hypoxia conditions [22]. VACV-based gene therapy has been shown to significantly inhibit cancer cell growth with low cytotoxic effects against healthy tissue in many different tumor types including myeloma [23], pancreatic carcinoma [24], hepatocellular carcinoma [25] and gastric carcinoma [26]. Despite its potential as a cancer therapy, the role of VACV-mediated *ING4* gene therapy for human pancreatic cancer has not been reported.

In this study, we constructed a novel oncolytic vaccinia virus expressing *ING4* (VV-*ING4*), and evaluated its therapeutic efficacy alone or in combination with gemcitabine against pancreatic cancer cells *in vitro* and *in vivo*. Our data implicate that VV-*ING4* is a potential candidate alone and in combination with gemcitabine for pancreatic cancer therapy.

## RESULTS

### Construction and characterization of VV-*ING4*

**Figure 1:**
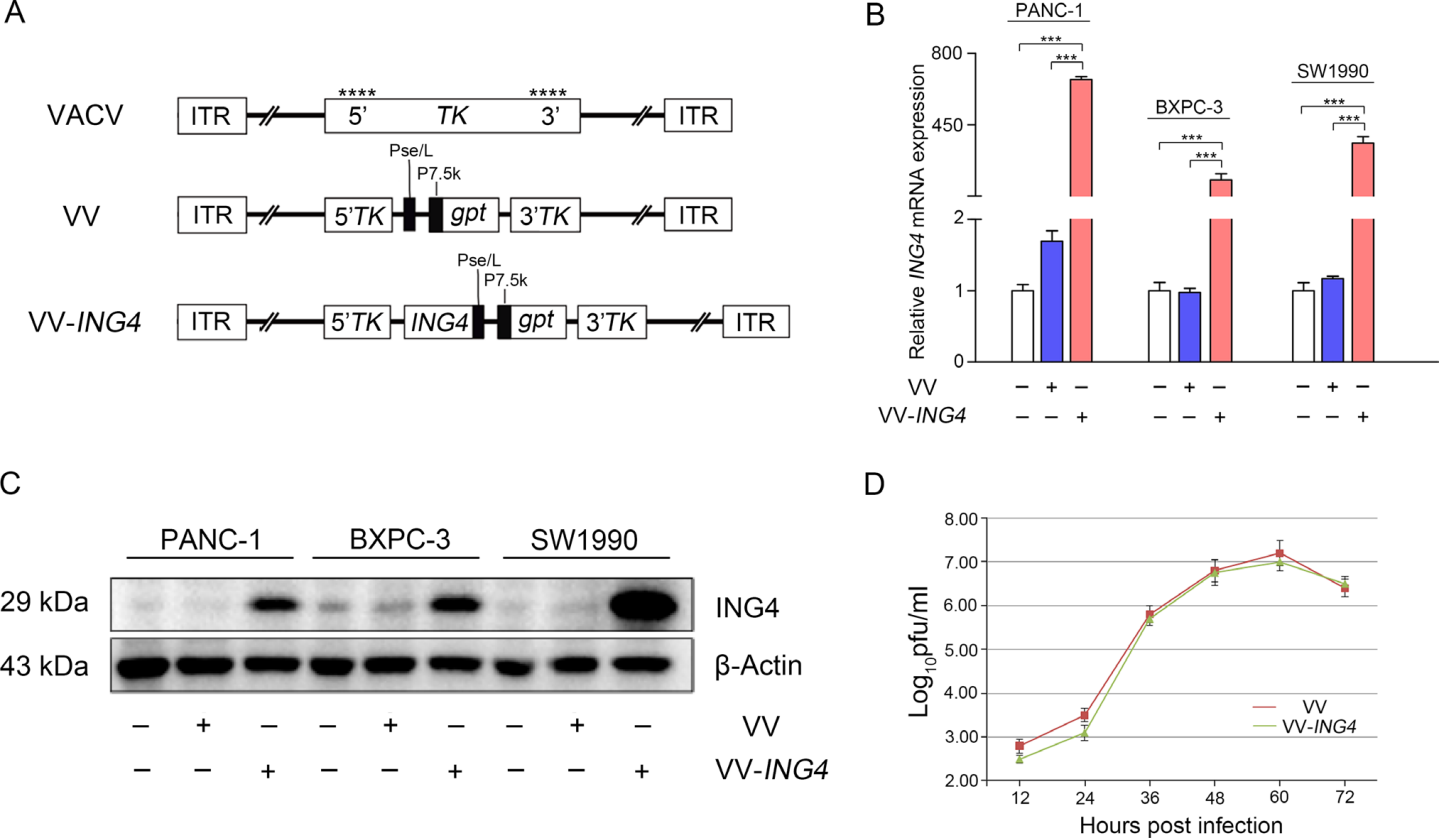
Characterization of VV-ING4 (**A**) Linear schematic of VV-*ING4* structure. VV: oncolytic vaccinia virus. *gpt* works as a screen gene. _****_, sites of anticipated homologous recombination. 5′TK and 3′TK, viral flanking sequences of the thymidine kinase gene. ITR, inverted terminal repeat. (**B**–**C**) The expression of *ING4*. Cells were infected with VV or VV-*ING4* at a MOI of 5 for 48 hrs. The *ING4* expression was determined using qPCR and western blot. GAPDH (B) and β-actin (C) served as internal controls. Data are presented as mean ± standard deviation (SD) or the representative of three separate experiments (*** represents *P* < 0.001). (**D**) Replication profiles of VV and VV-*ING4* in HEK293A cells. Cells were infected at a MOI of 0.02. The titer at each time point is presented as mean ± SD of at least three separate experiments.

We constructed an expression cassette consisting of the *ING4* gene and the *gpt* selection gene promoted by the viral promoter Pse/L or P7.5k, respectively. These genes were inserted into the viral *TK* gene (Figure 1A). VV, a control virus, was constructed similarly but without *ING4* gene insertion. The whole expression cassette was constructed into the pCB vector, which is a shuttle plasmid for VV and VV-*ING4* packaging. We verified significant expression of *ING4* gene in pancreatic cancer cells after infection with VV-*ING4* using qPCR (Figure 1B) and western blot (Figure 1C).

The titer and replication rate of each virus was measured. As shown in Table 1, VV and VV-*ING4* grew efficiently in HEK293A cells with expected and equivalent titers. The two recombinant viruses have similar replication rates (Figure 1D, *P* > 0.05).

**Table 1:** Virus particle titers and vp/pfu ratios

Recombinant vaccinia virus	Physical titer (vp/ml)	Infectious titer (pfu/ml)	Ratio (vp/pfu)
VV	5.03 × 10^9^	1.61 × 10^8^	31.2:1
VV-*ING4*	4.27 × 10^9^	1.76 × 10^8^	24.3:1

Note: vp, virus particle; pfu, plaque forming units.

### Pancreatic cancer-specific cytotoxicity of VV-*ING4 in vitro*

**Figure 2:**
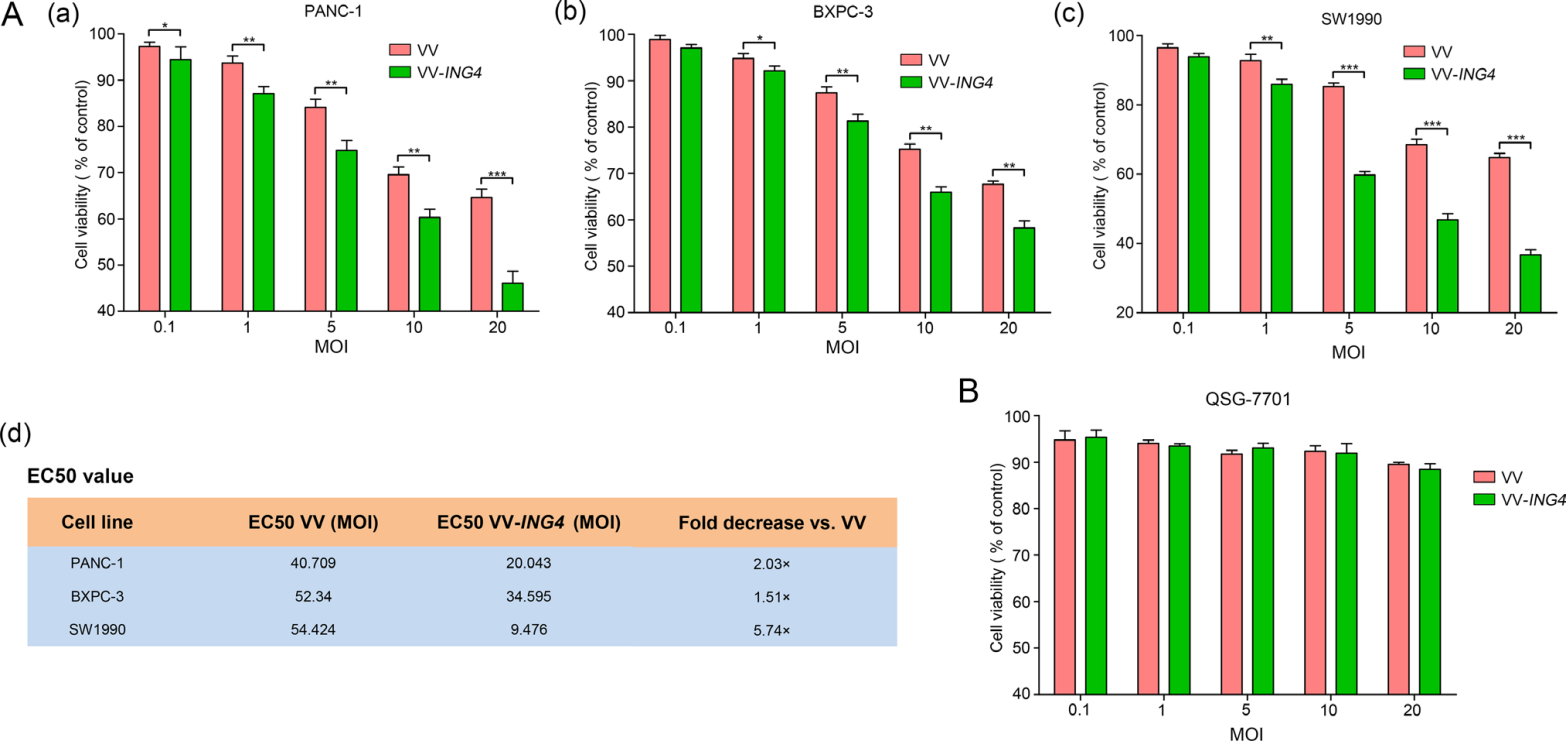
Pancreatic cancer-specific cytotoxicity of VV-ING4 Cells were seeded in 96-well plates and infected with VV or VV-*ING4* at a series of MOIs. Cell viability was determined by MTS cell proliferation assay at 48 hrs post-infection. (**A**) Cytotoxicity and EC50 of VV and VV-*ING4* in three pancreatic cancer cell lines. (**B**) Cytotoxicity of VV and VV-*ING4* in QSG-7701 cells. The results were presented as mean ± SD of three separate experiments (* represents *P* < 0.05, ** represents *P* < 0.01 and *** represents *P* < 0.001, one-way analysis of variance (ANOVA) and multiple comparisons).

The cytotoxicity of VV-*ING4* was evaluated in three pancreatic cancer cell lines (PANC-1, BXPC-3 and SW1990) and a normal liver cell line (QSG-7701) at 48 hrs post-infection using the MTS cell proliferation assay. The results showed that growth of VV-*ING4*-infected pancreatic cancer cells was inhibited in a dose-dependent manner, with EC50 values lower than that of VV (Figure 2A). However, growth of normal cells QSG-7701 was not inhibited significantly (Figure 2B). These findings indicate that VV-*ING4* efficiently represses *in vitro* pancreatic cancer cell growth with minimal influence on normal cells.

### VV-*ING4* induced apoptosis in pancreatic cancer cells *in vitro*

**Figure 3:**
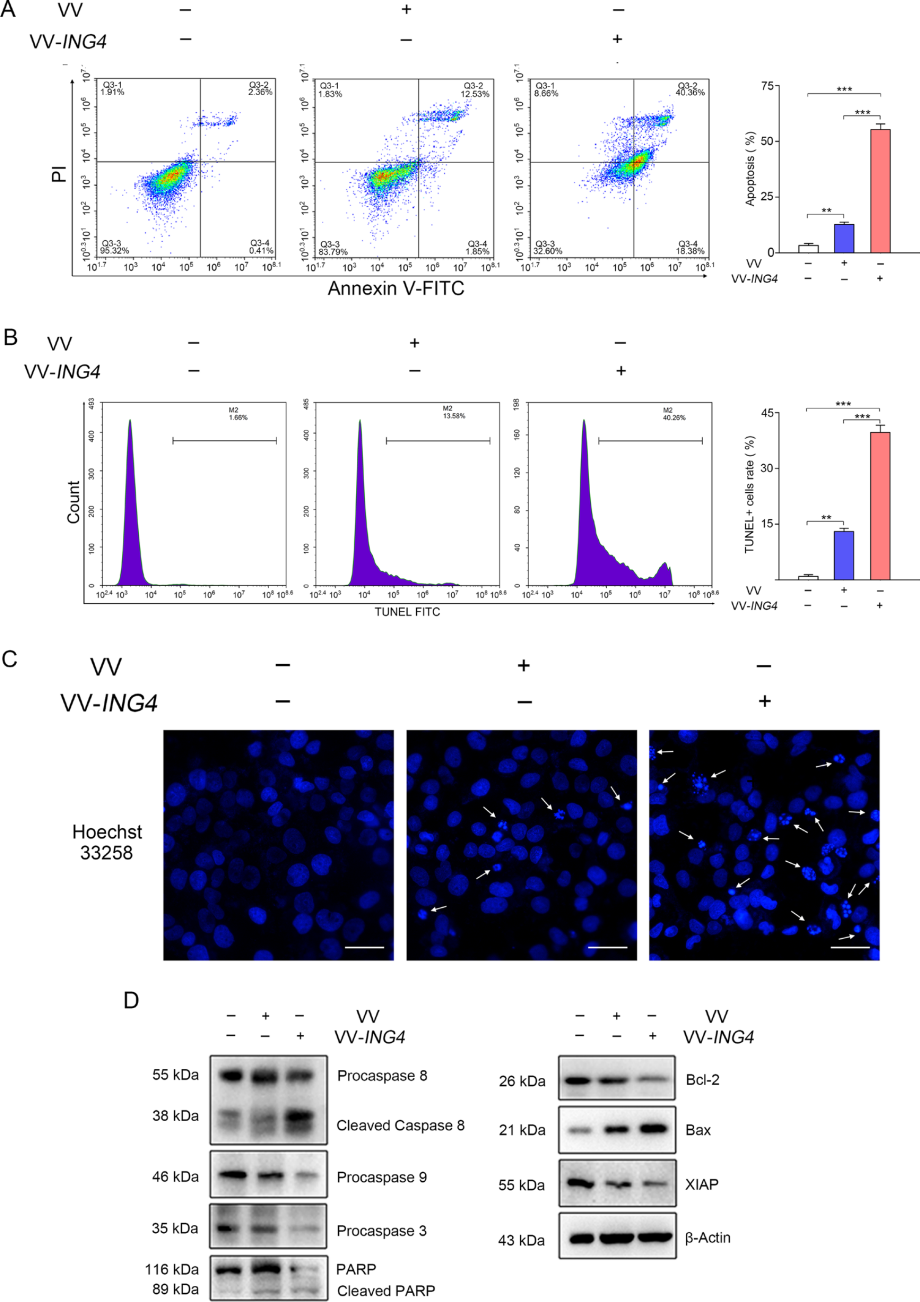
VV-ING4 induced apoptosis in pancreatic cancer cells *in vitro* (**A**) Apoptosis analysis using Annexin V-FITC/PI double staining. SW1990 cells were infected with VV or VV-*ING4* (MOI = 5) for 48 hrs. The florescence was analyzed by flow cytometry (Data are presented as mean ± SD of three separate experiments; ** represents *P* < 0.01, *** represents *P* < 0.001). (**B**–**C**) Apoptosis detection through measurement of DNA fragmentation. Cells were treated as indicated above, stained with TUNEL solution (B) or Hoechst 33258 (C). The arrows signify fragmented nucleic, scale bar: 500 μm; average values of TUNEL+ cells rate were determined by flow cytometry and expressed as mean ± SD of three separate experiments; ** represents *P* < 0.01, *** represents *P* < 0.001. (**D**) Apoptosis detection by Western blot. β-actin served as a loading control. Results are representative of three separate experiments.

We then examined whether VV-*ING4* inhibits cell growth by inducing apoptosis by treating pancreatic cancer cells with VV-*ING4* for 48 hrs. As shown in Figure 3A–3C, VV-*ING4* induced significant apoptosis in SW1990 cells, compared with VV- or PBS-treated cells. Western blot analysis revealed activation of Caspase 8/9/3 and enhanced poly (ADP-ribose) polymerase (PARP) cleavage, and Bax in VV-*ING4*-infected cells (Figure 3D). Expression of pro-survival proteins Bcl-2 and XIAP was remarkably decreased (Figure 3D). Taken together, these observations indicate that VV-*ING4* induces apoptosis in pancreatic cancer cells.

### VV-*ING4* induced G2/M cell cycle arrest in pancreatic cancer cells

**Figure 4:**
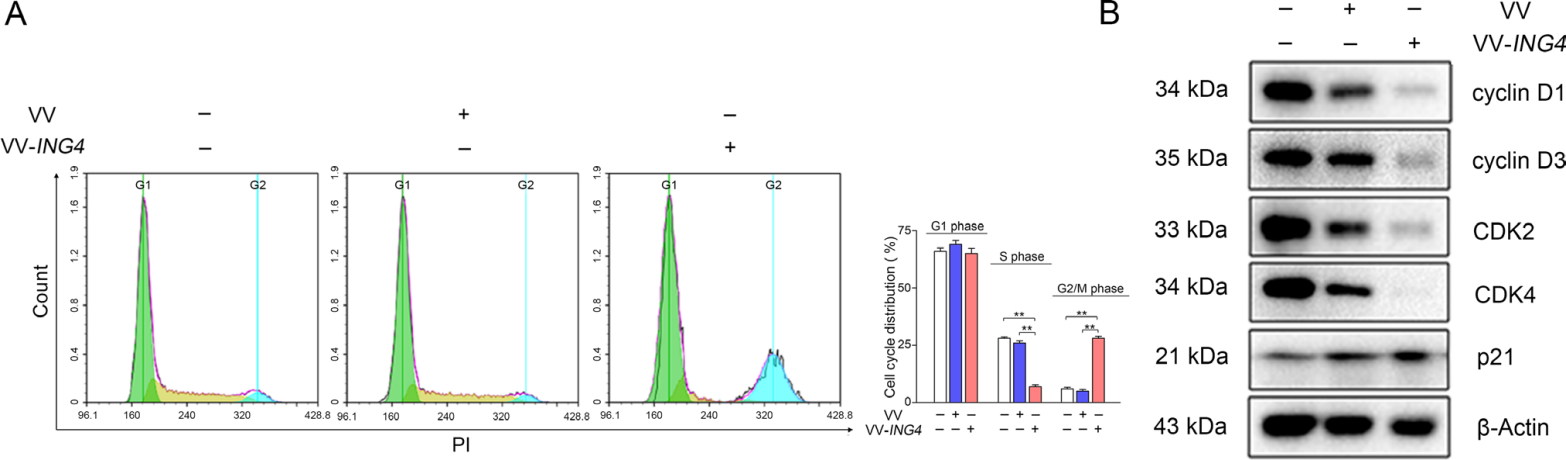
Regulation of the cell cycle by VV-ING4 in SW1990 cells (**A**) Analysis of cell cycle distribution. SW1990 cells were infected with VV or VV-*ING4* (MOI = 5) for 48 hrs. Cells were stained with PI. The florescence was analyzed by flow cytometry (Data are presented as mean ± SD of three separate experiments; ** represents *P* < 0.01). (**B**) Expression analysis of cell cycle-related proteins by western blot. Cells were infected as described above. β-actin was normalised as an internal reference. Data are representative of three determinations.

To assess the effect of VV-*ING4* on cell cycle regulation, SW1990 cells were treated as indicated above. The cells were then stained with Propidium Iodide (PI) and analyzed by flow cytometry. VV-*ING4*-treated cells contained a remarkably smaller S phase population and a significantly larger G2/M phase population than VV- or PBS-treated cells (Figure 4A). Western blot analysis showed that expression of cyclin D1, cyclin D3, CDK2 and CDK4 in VV-*ING4*-treated cells was significantly lower, whereas the expression of p21 was higher when compared with VV- or PBS-treated cells (Figure 4B). Taken together, these findings suggest that VV-*ING4* inhibits pancreatic cancer cells in part through alteration of cell cycle protein expression.

### Combination therapy of gemcitabine and VV-*ING4* synergistically suppress pancreatic cancer cell proliferation

**Figure 5:**
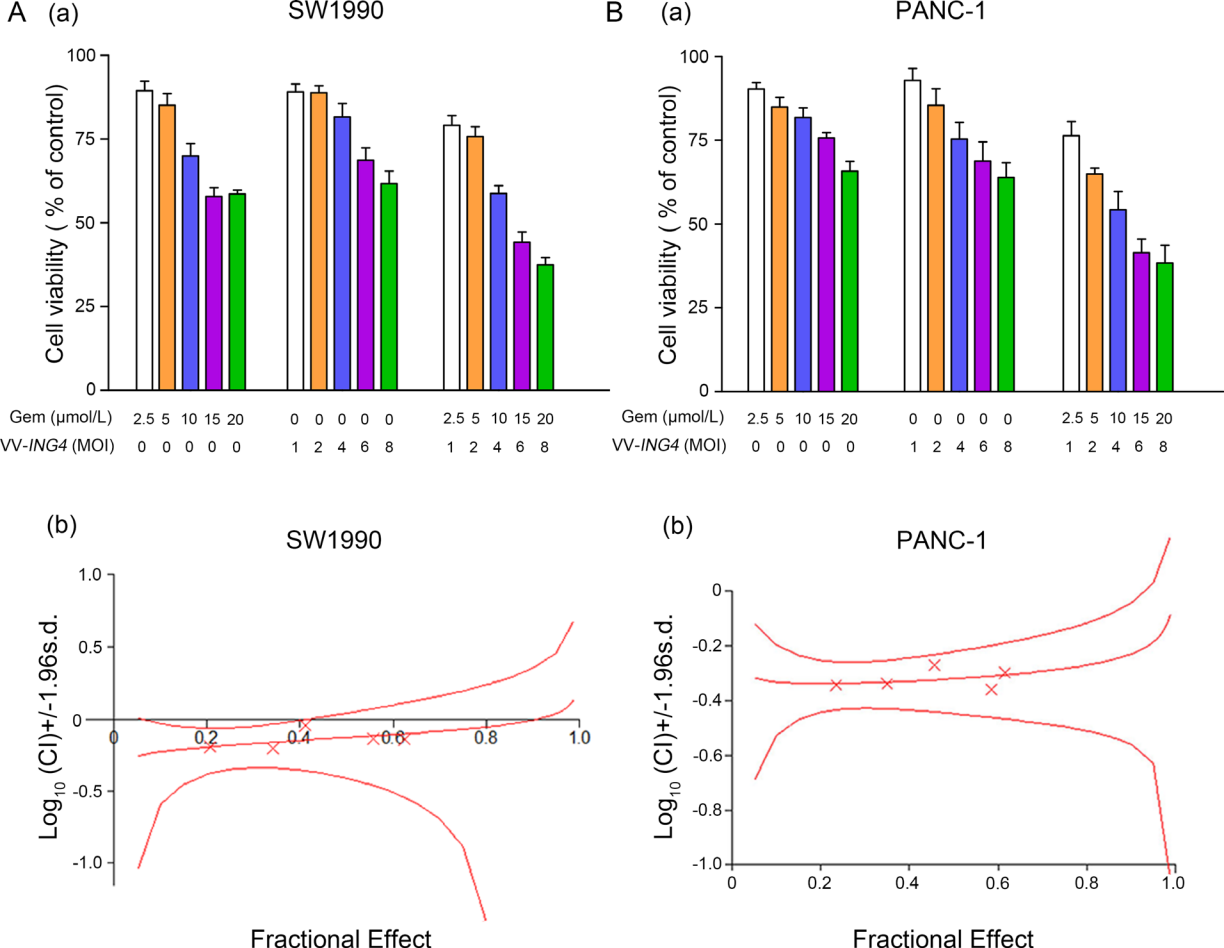
Evaluation of synergistic effect between gemcitabine and VV-ING4 *in vitro* (**A**) Dose–response histogram of cell viability. SW1990 and PANC-1 cells were seeded at 10,000 cells/well into 96-well plates. For synergism research, gemcitabine and VV-*ING4* were analyzed as a constant ratio of 2.5/1. Cell viability was measured 2 days later. Data shown are representative of three independent experiments. (**B**) Synergism detection was conducted by Chou–Talalay Combination Index (CI) analysis using CalcuSyn software (Biosoft, Cambridge Place, Cambrige, UK). The middle curve line stands for the simulated CI values, which were expressed as the log10 (CI) ± 1.96 SD. The log10 (CI) values represent an antagonism between the treatments when > 0, an additive efficiency when equal to 0 and a synergism when < 0.

To determine whether VV-*ING4* enhances the antitumor effects of gemcitabine, SW1990 and PANC-1 cells were treated as indicated in the MATERIALS AND METHODS section. Cell viability was determined 2 days later. As shown in Figure 5A, a negative dose-response effect was evident in all cases. Combination treatment significantly inhibited cell growth in both cell lines examined.

We evaluated the potential synergism between VV-*ING4* and gemcitabine by Chou–Talalay Combination Index (CI) analysis. At all the fractions considered, the Chou–Talalay CI was less than one in both SW1990 (CI 0.903–0.633, log10 (CI) < 0) and PANC-1 cells (CI 0.538–0.439, log10 (CI) < 0) (Figure 5B), indicating a potentiation effect of gemcitabine when combined with VV-*ING4*. Hence, the combined treatment of gemcitabine and VV-*ING4* synergistically represses pancreatic cancer cell proliferation.

### Combination therapy of gemcitabine and VV-*ING4* showed an enhanced tumor-killing effect *in vivo*

**Figure 6:**
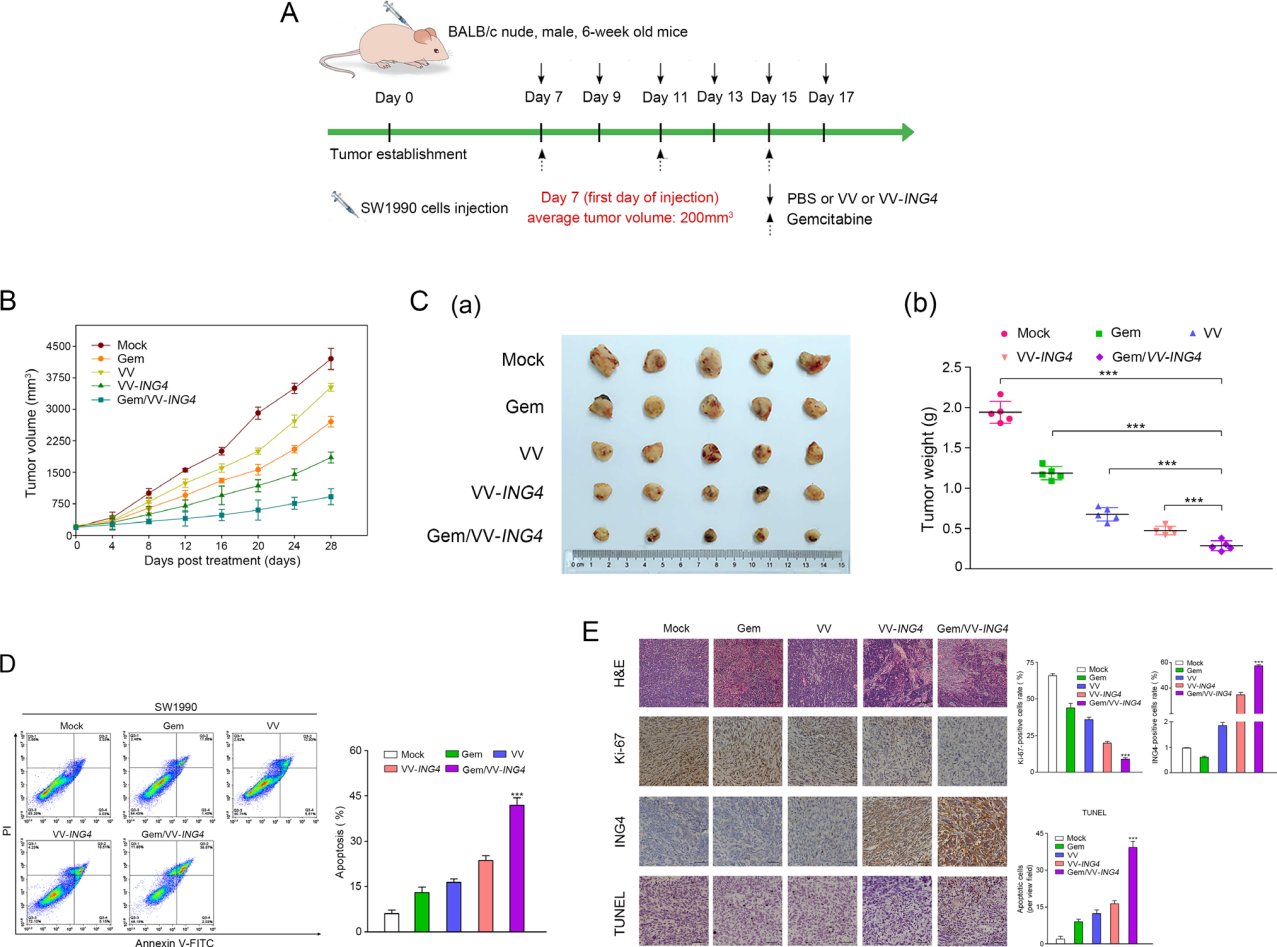
Combination therapy of gemcitabine and VV-ING4 showed an enhanced tumor-killing effect *in vivo* (**A**) Schematic of injections with experimental timeline. BALB/c athymic nude mice were intratumorally injected with PBS (100 μl), VV (1 × 10^7^ pfu) or VV-*ING4* (1 × 10^7^ pfu) every other day for a total of 6 times, or intraperitoneally injected with a single dose of gemcitabine (40 mg/Kg) every other three days for 3 times or a combination therapy based on gemcitabine and VV-*ING4*. The tumor volume (**B**) was determined. Mice were sacrificed at 4 weeks post-injection. Tumors were removed and weighted (**C**). Tumor cell apoptosis *in vivo* was detected by flow cytometry (**D**) (*** represents *P* < 0.001. Results are presented as mean ± SD of three separate experiments). (**E**) Histopathological analysis. Tumor samples from different groups were subjected to H&E staining, immunohistochemical analysis and TUNEL assay. Quantitative graph for percentage of Ki67, ING4 and TUNEL positive cells are given (5 random fields, 4 sections for each sample, *** represents *P* < 0.001, scale bars: 500 μm, 400× magnification).

To analyze the impact of the combined treatment *in vivo*, animal experiments (BALB/c athymic nude mice) were performed following the protocol described in Figure 6A. We found that mean tumor volume rapidly declined in animals treated with the combination therapy when compared to tumors from animals treated with either individual therapy (Figure 6B–6C). To affirm the underlying mechanism, we evaluated the effect of combined treatment on tumor cell apoptosis *in vivo* by flow cytometry analysis. As shown in Figure 6D, the percentage of apoptotic SW1990 cells was significantly higher in the combination treatment group, compared with either individual treatment group.

Hematoxylin and eosin (H&E) and immunohistochemical (IHC) staining for Ki-67 were applied to assess and compare tumor tissue histopathology. Decreased Ki-67 expression and H&E staining revealed that combination therapy had a more cytotoxic effect on tumor proliferation than either treatment alone (Figure 6E). IHC analysis using anti-ING4 antibody confirmed expression of *ING4* in the tumor tissues following by combined treatment (Figure 6E).

Apoptosis was further examined using the terminal deoxynucleotidyl transferase-mediated dUTP nick end labeling (TUNEL) assay. Results from this experiment showed significantly higher apoptosis rates in the combination treatment when compared with either individual treatment (Figure 6E).

## DISCUSSION

In this study, we constructed an oncolytic vaccina virus expressing tumor suppressor *ING4* (VV-*ING4*) and revealed that *ING4* expression exhibits anti-tumor effects alone and in combination with gemcitabine against pancreatic cancer cells *in vitro* and *in vivo*.

*ING4* expression induced significant apoptosis in SW1990 human pancreatic cancer cells, consistent with the findings of previous studies [12, 27, 28]. Chen *et al.* showed miR-214 inhibits apoptosis in pancreatic cancer tissues by downregulating *ING4* expression [29]. In this study, we found that infection of VV-*ING4* reduced the ratio of Bcl-2/Bax and induced the activation of Caspase 8/9/3 and PARP in SW1990 human pancreatic cancer cells (Figure 3D), suggesting that Caspase-dependent apoptosis and the Bcl-2 family is participated in VV-*ING4*-induced cytotoxicity. This finding is in line with a previous study which identified the Fas/Caspase-8 pathway as a mechanism of ING4-induced apoptosis in human melanoma cells [30]. Another potential mechanism by which ING4 induces apoptosis is promotion of p53. An earlier study by Shiseki *et al.* reported that ING4 induced apoptosis of RKO colon cancer cells via enhancing the function of p53 [11]. The tumor suppressor p53 is well known to have a pro-apoptotic function through regulation of Bcl-2 and Bax expression [31, 32]. These results support the involvement of Bcl-2 family proteins in ING4-induced apoptosis [33]. ING4 may also induce apoptosis through negative regulation of NF-κB signaling [27] and via cooperating with other tumor suppressors [34]. More research is needed to tease out the mechanism underlying ING4 pro-apoptotic effects.

We also found that *ING4* expression induces significant cell cycle arrest in pancreatic cancer cells. This is consistent with previous studies that have shown that ING4 promotes cell cycle arrest in other cancer cells [14, 28]. It is well known that cyclins, cyclin-dependent kinases (CDKs) and CDK inhibitors are crucial for cell cycle progression. The activities of various cyclin/CDK complexes are inhibited by the CDK inhibitors, such as p16, p21, and p27. It has been proved that ING4 upregulated p21 in a p53-dependent manner [14]. p21 restricts the activity of cyclin E-CDK2, cyclin D3-CDK4 and cyclin B1/CDC2 complexes leading to G2/M arrest [35, 36]. p21 also inhibits cyclin D1 activity [37], which can cause G2/M arrest [38, 39]. Interestingly, ING4 was reported to connect with p300 [11] and NF-κB subunit p65 (RelA) [8]. Whether ING4-induced upregulation of p21 was related with these p53-independent signaling pathways still needs to be further studied. In our study, flow cytometry analysis showed VV-*ING4* infection altered the cell cycle with S-phase reduction and G2/M phase arrest, which differs from the preceding report using RKO colon cancer cells [11]. This may be due to the use of different tumor cell lines with specific characteristics in these studies. Further study indicated that ING4 expression significantly decreased the expression of cyclin D1, cyclin D3, CDK2 and CDK4, whereas the expression of p21 was increased. Therefore, ING4-induced upregulation of p21 may contribute to the G2/M cell cycle arrest in VV-*ING4*-treated human pancreatic cancer cells.

Finally, combination therapy of VV-*ING4* and gemcitabine synergistically promoted cell death in pancreatic cancer cells *in vitro* and *in vivo*. Multiple mechanisms may account for this improved anti-tumor efficiency. Xu *et al.* reported that ING4 enhances the sensitivity of cells to chemotherapy by decreasing p-Stat3 expression [40]. In addition, cooperative regulation of apoptosis pathways and the inhibition of tumor angiogenesis between IGN4 and chemotherapy may also contribute to the synergistic effect [15]. Gemcitabine is a standard first-line treatment for pancreatic cancer. However, one disadvantage to gemcitabine efficacy is its disappointing penetration into tumor parenchyma [41]. VV-*ING4* may selectively replicate in and lyse tumor cells, which disrupt tumor architecture thus facilitating gemcitabine penetration to generate a synergistic effect. Additionally, gemcitabine has been shown to enhance vaccine efficacy by obviating CD11b^+^/Gr-1^+^ myeloid-derived suppressor cells (MDSCs) in a murine model of pancreatic carcinoma [42], and vice versa [43]. In addition, suboptimal doses of gemcitabine invigorate viral uptake in pancreatic cancer cell lines [44]. Furthermore, the synergism in the combination therapy may also be caused by unblocking host pathways, transporting viruses with greater efficiency and/or increasing virus generation at the tumor site [45]. In this study, flow cytometry analysis and TUNEL assay indicate that the percentage of apoptotic cells in the combined therapy group was significantly higher compared with either individual sample, exhibiting an apoptosis-induction synergism, which is consistent with the previous study [15].

To our knowledge, this is the first study to show that VV-*ING4* can induce the death of pancreatic cancer cells and in combination with gemcitabine shows a synergistic effect *in vitro* and *in vivo*. However, there still remain many obstacles to be conquered for in applying VV-*ING4* to clinical practice, such as efficient delivery and expression of *ING4*, the cytotoxicity of viral vector, or immune responses against viral antigens. Hence, future studies aim to develop a tumor-specific delivery system using carrier cells or nanotechnologies to promote the transfection efficiency of VV-*ING4* and weaken the potential systemic toxicity in our future work.

In conclusion, our data suggest that VV-*ING4* is a conceivable candidate alone and in combination with gemcitabine for pancreatic cancer therapy.

## MATERIALS AND METHODS

### Cell lines and vectors

The human embryonic kidney cell line 293A and the liver cell line QSG-7701 were retained in our lab. SW1990, BXPC-3 and PANC-1 human pancreatic cancer cell lines were purchased from the Cell Bank of the Chinese Academy of Sciences (Shanghai, China). The cell lines were authenticated by short-tandem repeat profiling. Cells were cultivated in Dulbecco’s modified Eagle’s medium (DMEM), supplied with 10% fetal bovine serum (FBS), penicillin (100 U/ml), streptomycin (100 μg/ml), and L-glutamine (2 mM, Gibco, Invitrogen), incubated at 37°C in a humidified 5% CO2 atmosphere in air. The wild-type vaccinia virus (VACV) and pCB vector were kindly offered by academician Xinyuan Liu.

### Regents

Cell cycle and apoptosis detection kit and the TUNEL kit were purchased from Beyotime biotechnology company (Beyotime, Jiangsu, China). Tissue dissociation kit was purchased from Miltenyi (Miltenyi, Bergisch Gladbach, Germany). Antibodies against ING4 (1:1000), Bax (1:1000), cyclin D1 (1:5000), cyclin D3 (1:5000), CDK2 (1:5000) and CDK4 (1:5000) were purchased from Abcam (Shanghai, China). Antibodies against Caspase 8 (1:1000), Caspase 3 (1:1000) and Bcl-2 (1:1000) were obtained from the Bioworld Company (Minnesota, USA). Caspase 9 (1:1000), XIAP (1:1000) and p21 (1:1000) antibodies were purchased from EMD Millipore Corporation (Billerica, MA, USA). PARP antibody (1:1000) was purchased from Sino Biological Inc. (Beijing, China). β-actin (1:5000) antibody was obtained from HuaAn Biotechnology Co., Ltd. (Hangzhou, China). The primers used in this study were synthesized in Generay Biotech Co.,Ltd (Shanghai, China).

### Construction, identification and purification of oncolytic vaccinia viruses

The entire *ING4* cDNA sequence was PCR-amplified with specific primer pairs: 5′-GGCCTCGAGATGGCTGCGGGGATGTATTTG-3′ [forward] and 5′-GGCGGTACCCTATTTCTTCTTCCGTTCTTGGGAG- 3′ [reverse]. The artificial DNA was digested with BglII and ECORI (Takara, Japan), then inserted into pCB plasmid to create pCB-*ING4*. After sequence confirmation, pCB or pCB-*ING4* homologously recombined with VACV in HEK293A cells using Lipofectamine 3000 (Invitrogen, Shanghai, China). After observing cytopathic effect, cell culture medium was gathered to obtain recombinant viruses. Mycophenolic acid, dioxopurine and hypoxanthine were applied to get rid of VACV. Recombinant vaccinia viruses were amplified in HEK293A cells and purified by ultracentrifugation. OD260 assay and plaque formation assay were used to determine viral titers.

### Viral replication rate

Viral growth curves were determined as described previously [46]. Briefly, HEK293A cells at a density of 62,500/cm^2^ were infected with VV or VV-*ING4* at a MOI of 0.02. At 12 hrs intervals post-infection, the supernatant was collected and titered by plaque assay in HEK293A cells at a density of 50,000/cm^2^. Virus titers were expressed as plaque forming units (pfu) per mL (pfu/ml).

### qPCR

RNA was extracted with Trizol reagent (Invitrogen, Shanghai, China) following the manufacturer’s instructions. cDNA was generated using the PrimeScript RT reagent kit (Tokyo, Japan). The qPCR reactions were conducted in a total volume of 20 μl by using the following procedure: 1 cycle at 95°C (10 min), then 60°C (30 sec), followed by 39 cycles at 95°C (10 sec), 60°C (30 sec). PCR amplicons were determined based on SYBR Green I detection (Roche Diagnostic, Indianapolis, USA), and the authenticity was certified by melting curve analysis. Quantitative PCR was operated using the CFX-96 qPCR system and iQ SYBR Green Supermix (Bio-Rad). Relative gene expression was determined via the 2^-ΔΔCt^ method. The primers used are as follows: Gene: Sequence; *ING4:* 5′-AGTATGGGATGCCCTCAGTG-3′ (forward), 5′-GACCTGGTGACAAAGGCAAT-3′ (reverse);*GAPDH:*5′-CTTTGGTATCGTGGAAGGACTC-3′ (forward), 5′-GTAGAGGCAGGGGATGATGTTCT-3′ (forward).

### Western blot analysis

Floating and adherent cells were harvested in lysis buffer (Beyotime, Jiangsu, China) involving 1% Complete Mini-Protease Inhibitor Cocktail (Roche Diagnosis, Switzerland), and 5 mM NaF. Protein extractions were quantified using the BCA kit (Thermo scientific, MA, USA) and heated for 10 min at 100°C. 30 μg of protein was resolved in 12% SDS-PAGE and transferred to nitrocellulose membrane (Merk Millipore, Germany). After blocked for 1 hour at 37°C, the membranes were immunobloted with different antibodies overnight at 4°C. Membranes were then washed with TBST and incubated with HRP-conjugated goat anti-rabbit or anti-mouse antibody (1:5000) for 1 hour at room temperature. Finally, blots were detected using ChemiDoc™ MP Imaging System (Bio-Rad) with a SuperEnhanced chemiluminescence detection kit (Applygen, Beijing, China).

### MTS cell proliferation assay

PANC-1, BXPC-3 and SW1990 cells and normal liver cells QSG-7701 were dispensed in 96-well culture plates at a density of 1 × 10^4^ cells/well. After attachment, cells were infected with VV or VV-*ING4* at a MOI of 0.1, 1, 5, 10 or 20. The medium added with PBS was a blank control. At 48 hrs post-infection, cells were further incubated with 20 µl/well MTS reagent (Promega, Madison, WI, USA) at 37°C for 4 hrs. After shaked for 1 min, plates were read at 490 nm using a Microplate Reader Model 550 (Bio-Rad, Shanghai, China). Cell viability% = [(experimental group OD - blank control OD)/(negative control OD - blank control OD)] × 100%. The viral MOIs required to kill 50% of cells (the median effective concentration [EC50]) were determined.

### Hoechst DNA staining

SW1990 cells at a density of 2 × 10^4^ cells/coverslip were treated with VV or VV-*ING4* at a MOI of 5. At 48 hrs post-infection, cells were fixed with 4% paraformaldehyde, stained with Hoechst 33258 (Beyotime, Jiangsu, China, 1 µg/ml) for 15 min at room temperature. The chromatin changes were visualized under a Nikon standard fluorescence microscope (Ti-U, Nikon, Japan) with NIS-Elements BR 4.50.00 imaging software. Four random microscopic fields (400× magnification) were selected per sample. Three replicates were performed.

### Flow cytometry analysis

At 48 hrs post-infection, SW1990 cells were harvested at a density of 6 × 10^5^ cells/ml. 2 ml collected cells were centrifuged, incubated in 300 µl of 1X binding buffer with 5 µl of Annexin V-FITC and 10 µl of PI for 10 min. For TUNEL assay, 2 ml collected cells were fixed in 4% paraformaldehyde solution for 30 min and permeabilizated in 0.1% Triton X-100 solution for 10 min. Cells were incubated in TUNEL reaction mixture (50 μl) for 60 min at 37°C, then transferred into a tube to a total volume of 500 μl in PBS. The florescence was analyzed by NovoCyte Flow Cytometer (ACEA Biosciences Inc. San Diego, CA).

For cell cycle analysis, 1 ml collected cells were centrifuged, fixed with cold 75% alcohol overnight, incubated in 500 µl of staining buffer containing 25 µl of PI and 10 µl of RNase A for 30 min. The florescence was analyzed by flow cytometry.

### Dose–response and combination index analysis

SW1990 and PANC-1 cells in 96-well plates were treated with different doses of gemcitabine (µM) (Eli Lilly, Indianapolis, USA) or VV-*ING4* (MOI), or combination with a constant ratio of gemcitabine/VV-*ING4* of 2.5/1. Cell viability was measured 48 hrs later, based on which the synergism between gemcitabine and VV-*ING4* was analyzed with Calcusyn Software (Biosoft, Cambridge, UK). For combination index plots, CI is expressed as the log10 (CI) ± 1.96 SD, the 95% confidence intervals are shown where estimable. CI values were calculated over a scope of levels of growth inhibition (GI) from 20% to 80% of the fraction affected.

### Animal studies

All animal experiments were approved by the Institutional Animal Care and Use Committee, performed according to the Guide for the Care and Use of Laboratory Animals of the National Institutes of Health, and followed the guidelines of the Animal Welfare Act. BALB/c athymic nude mice (male, 5-week old and 17–20 g) were purchased from Shanghai Experimental Animal Center (Shanghai, China). One week later, mice were randomly grouped (7 mice per group) and subcutaneously injected on the neck with 1 × 10^6^ SW1990 cells. When tumors grew up to about 200 mm^3^, mice were injected following the schematic of injections in Figure 6A. On day 7 post-intervention, xenografted tumors from two mice per group were digested enzymatically for apoptosis detection. Tumor volumes were calculated every other three days. Mice were sacrificed at 4 weeks post-injection according to ethical instructions. Tumors were separated, fixed by 4% paraformaldehyde, imbedded in paraffin, finally cut into 4 µm sections for hematoxylin and eosin staining, immunohistochemistry analysis and TUNEL assay according to manufacturers’ instructions. For immunohistochemistry analysis, slides were incubated with primary antibodies: anti-Ki 67 (1:100, HuaAn, Hangzhou, China) or anti-ING4 (1:100, Abcam, Shanghai, China) overnight at 4°C, then incubated with biotinylated secondary antibody, further visualized using a diaminobenzidine (DAB) Kit (Thermo scientific, Waltham, MA).

### Statistical analysis

An analysis of variance (ANOVA) was applied for comparison of 3 or more groups. The analysis of the combined effects was performed with CalcuSyn software 2.0 (Biosoft, Cambridge, UK). Data are expressed as mean ± SD. Statistical analysis was performed with IBM SPSS Statistics software version 20 (SPSS Inc., Chicago, IL). Statistical significance was prescribed at *P* < 0.05.

## Abbreviations

*ING4*: inhibitor of growth family member 4
VV: oncolytic vaccinia virus
MOI: multiplicity of infection
PBS: phosphate buffered saline

## References

[1] Ferlay J, Soerjomataram I, Dikshit R, Eser S, Mathers C, Rebelo M, Parkin DM, Forman D, Bray F (2015). Cancer incidence and mortality worldwide: sources, methods and major patterns in GLOBOCAN 2012. Int J Cancer.

[2] Beuran M, Negoi I, Paun S, Ion AD, Bleotu C, Negoi RI, Hostiuc S (2015). The epithelial to mesenchymal transition in pancreatic cancer: A systematic review. Pancreatology.

[3] Warshaw AL, Gu ZY, Wittenberg J, Waltman AC (1990). Preoperative staging and assessment of resectability of pancreatic cancer. Arch Surg.

[4] Alberts SR, Gores GJ, Kim GP, Roberts LR, Kendrick ML, Rosen CB, Chari ST, Martenson JA (2007). Treatment options for hepatobiliary and pancreatic cancer. Mayo Clin Proc.

[5] Zhou Y, Shou F, Zhang H, You Q (2016). Adenovirus-delivered wwox inhibited lung cancer growth *in vivo* in a mouse model. Cancer Gene Ther.

[6] Wu J, Zhu Y, Xu C, Xu H, Zhou X, Yang J, Xie Y, Tao M (2016). Adenovirus-mediated p53 and ING4 gene co-transfer elicits synergistic antitumor effects through enhancement of p53 acetylation in breast cancer. Oncol Rep.

[7] Soliman MA, Riabowol K (2007). After a decade of study-ING, a PHD for a versatile family of proteins. Trends Biochem Sci.

[8] Garkavtsev I, Kozin SV, Chernova O, Xu L, Winkler F, Brown E, Barnett GH, Jain RK (2004). The candidate tumour suppressor protein ING4 regulates brain tumour growth and angiogenesis. Nature.

[9] Berger PL, Frank SB, Schulz VV, Nollet EA, Edick MJ, Holly B, Chang TT, Hostetter G, Kim S, Miranti CK (2014). Transient induction of ING4 by Myc drives prostate epithelial cell differentiation and its disruption drives prostate tumorigenesis. Cancer Res.

[10] Ozer A, Wu LC, Bruick RK (2005). The candidate tumor suppressor ING4 represses activation of the hypoxia inducible factor (HIF). Proc Natl Acad Sci USA.

[11] Shiseki M, Nagashima M, Pedeux RM, Kitahama-Shiseki M, Miura K, Okamura S, Onogi H, Higashimoto Y, Appella E, Yokota J, Harris CC (2003). p29ING4 and p28ING5 bind to p53 and p300, and enhance p53 activity. Cancer Res.

[12] Cai L, Li X, Zheng S, Wang Y, Wang Y, Li H, Yang J, Sun J (2009). Inhibitor of growth 4 is involved in melanomagenesis and induces growth suppression and apoptosis in melanoma cell line M14. Melanoma Res.

[13] Xie Y, Zhang H, Sheng W, Xiang J, Ye Z, Yang J (2008). Adenovirus-mediated ING4 expression suppresses lung carcinoma cell growth via induction of cell cycle alteration and apoptosis and inhibition of tumor invasion and angiogenesis. Cancer Lett.

[14] Zhang X, Xu LS, Wang ZQ, Wang KS, Li N, Cheng ZH, Huang SZ, Wei DZ, Han ZG (2004). ING4 induces G2/M cell cycle arrest and enhances the chemosensitivity to DNA-damage agents in HepG2 cells. FEBS Lett.

[15] Xie Y, Sheng W, Miao J, Xiang J, Yang J (2011). Enhanced antitumor activity by combining an adenovirus harboring ING4 with cisplatin for hepatocarcinoma cells. Cancer Gene Ther.

[16] Kim S, Chin K, Gray JW, Bishop JM (2004). A screen for genes that suppress loss of contact inhibition: identification of ING4 as a candidate tumor suppressor gene in human cancer. Proc Natl Acad Sci USA.

[17] Li M, Jin Y, Sun WJ, Yu Y, Bai J, Tong DD, Qi JP, Du JR, Geng JS, Huang Q, Huang XY, Huang Y, Han FF (2009). Reduced expression and novel splice variants of ING4 in human gastric adenocarcinoma. J Pathol.

[18] Buller RM, Smith GL, Cremer K, Notkins AL, Moss B (1985). Decreased virulence of recombinant vaccinia virus expression vectors is associated with a thymidine kinase-negative phenotype. Nature.

[19] Worschech A, Haddad D, Stroncek DF, Wang E, Marincola FM, Szalay AA (2009). The immunologic aspects of poxvirus oncolytic therapy. Cancer Immunol Immunother.

[20] Poland GA, Grabenstein JD, Neff JM (2005). The US smallpox vaccination program: a review of a large modern era smallpox vaccination implementation program. Vaccine.

[21] Carroll MW, Moss B (1997). Poxviruses as expression vectors. Curr Opin Biotechnol.

[22] Hiley CT, Yuan M, Lemoine NR, Wang Y (2010). Lister strain vaccinia virus, a potential therapeutic vector targeting hypoxic tumours. Gene Ther.

[23] Lei W, Wang S, Yang C, Huang X, Chen Z, He W, Shen J, Liu X, Qian W (2016). Combined expression of miR-34a and Smac mediated by oncolytic vaccinia virus synergistically promote anti-tumor effects in Multiple Myeloma. Sci Rep.

[24] Chard LS, Lemoine NR, Wang Y (2015). New role of Interleukin-10 in enhancing the antitumor efficacy of oncolytic vaccinia virus for treatment of pancreatic cancer. OncoImmunology.

[25] Pan Q, Huang Y, Chen L, Gu J, Zhou X (2014). SMAC-armed vaccinia virus induces both apoptosis and necroptosis and synergizes the efficiency of vinblastine in HCC. Hum Cell.

[26] Jun KH, Gholami S, Song TJ, Au J, Haddad D, Carson J, Chen CH, Mojica K, Zanzonico P, Chen NG, Zhang Q, Szalay A, Fong Y (2014). A novel oncolytic viral therapy and imaging technique for gastric cancer using a genetically engineered vaccinia virus carrying the human sodium iodide symporter. J Exp Clin Cancer Res.

[27] Li M, Zhu Y, Zhang H, Li L, He P, Xia H, Zhang Y, Mao C (2014). Delivery of inhibitor of growth 4 (ING4) gene significantly inhibits proliferation and invasion and promotes apoptosis of human osteosarcoma cells. Sci Rep.

[28] Xie YF, Sheng W, Xiang J, Zhang H, Ye Z, Yang J (2009). Adenovirus-mediated ING4 expression suppresses pancreatic carcinoma cell growth via induction of cell-cycle alteration, apoptosis, and inhibition of tumor angiogenesis. Cancer Biother Radiopharm.

[29] Zhang XJ, Ye H, Zeng CW, He B, Zhang H, Chen YQ (2010). Dysregulation of miR-15a and miR-214 in human pancreatic cancer. J Hematol Oncol.

[30] Ma Y, Cheng X, Wang F, Pan J, Liu J, Chen H, Wang Y, Cai L (2016). ING4 inhibits proliferation and induces apoptosis in human melanoma A375 cells via the Fas/Caspase-8 apoptosis pathway. Dermatology.

[31] Basu A, Haldar S (1998). The relationship between BcI2, Bax and p53: consequences for cell cycle progression and cell death. Mol Hum Reprod.

[32] Chipuk JE, Kuwana T, Bouchier-Hayes L, Droin NM, Newmeyer DD, Schuler M, Green DR (2004). Direct activation of Bax by p53 mediates mitochondrial membrane permeabilization and apoptosis. Science.

[33] Li X, Zhang Q, Cai L, Wang Y, Wang Q, Huang X, Fu S, Bai J, Liu J, Zhang G, Qi J (2009). Inhibitor of growth 4 induces apoptosis in human lung adenocarcinoma cell line A549 via Bcl-2 family proteins and mitochondria apoptosis pathway. J Cancer Res Clin Oncol.

[34] Nagahama Y, Ishimaru M, Osaki M, Inoue T, Maeda A, Nakada C, Moriyama M, Sato K, Oshimura M, Ito H (2008). Apoptotic pathway induced by transduction of RUNX3 in the human gastric carcinoma cell line MKN-1. Cancer Sci.

[35] Bates S, Ryan KM, Phillips AC, Vousden KH (1998). Cell cycle arrest and DNA endoreduplication following p21Waf1/Cip1 expression. Oncogene.

[36] Abbas T, Dutta A (2009). p21 in cancer: intricate networks and multiple activities. Nat Rev Cancer.

[37] Vermeulen K, Van Bockstaele DR, Berneman ZN (2003). The cell cycle: a review of regulation, deregulation and therapeutic targets in cancer. Cell Prolif.

[38] Lahti JM, Li H, Kidd VJ (1997). Elimination of cyclin D1 in vertebrate cells leads to an altered cell cycle phenotype, which is rescued by overexpression of murine cyclins D1, D2, or D3 but not by a mutant cyclin D1. J Biol Chem.

[39] Khleif SN, DeGregori J, Yee CL, Otterson GA, Kaye FJ, Nevins JR, Howley PM (1996). Inhibition of cyclin D-CDK4/CDK6 activity is associated with an E2F-mediated induction of cyclin kinase inhibitor activity. Proc Natl Acad Sci USA.

[40] Cao L, Chen S, Zhang C, Chen C, Lu N, Jiang Y, Cai Y, Yin Y, Xu J (2015). ING4 enhances paclitaxel’s effect on colorectal cancer growth *in vitro* and *in vivo*. Int J Clin Exp Pathol.

[41] Huxham LA, Kyle AH, Baker JH, Nykilchuk LK, Minchinton AI (2004). Microregional effects of gemcitabine in HCT-116 xenografts. Cancer Res.

[42] Ishizaki H, Manuel ER, Song GY, Srivastava T, Sun S, Diamond DJ, Ellenhorn JD (2011). Modified vaccinia Ankara expressing survivin combined with gemcitabine generates specific antitumor effects in a murine pancreatic carcinoma model. Cancer Immunol Immunother.

[43] Al Yaghchi C, Zhang Z, Alusi G, Lemoine NR, Wang Y (2015). Vaccinia virus, a promising new therapeutic agent for pancreatic cancer. Immunotherapy.

[44] Bhattacharyya M, Francis J, Eddouadi A, Lemoine NR, Halldén G (2011). An oncolytic adenovirus defective in pRb-binding (dl922-947) can efficiently eliminate pancreatic cancer cells and tumors *in vivo* in combination with 5-FU or gemcitabine. Cancer Gene Ther.

[45] Nguyen TL, Tumilasci VF, Singhroy D, Arguello M, Hiscott J (2009). The emergence of combinatorial strategies in the development of RNA oncolytic virus therapies. Cell Microbiol.

[46] Bonaldo MC, Garratt RC, Marchevsky RS, Coutinho ES, Jabor AV, Almeida LF, Yamamura AM, Duarte AS, Oliveira PJ, Lizeu JO, Camacho LA, Freire MS, Galler R (2005). Attenuation of recombinant yellow fever 17D viruses expressing foreign protein epitopes at the surface. J Virol.

